# Testicular Seminoma Presenting with Bilateral Blindness: Looking Beyond the Eye

**DOI:** 10.7759/cureus.7534

**Published:** 2020-04-04

**Authors:** Rahul Myadam, Ashraf Gohar

**Affiliations:** 1 Internal Medicine, University of Missouri-Kansas City, Kansas City, USA; 2 Pulmonary and Critical Care, Truman Medical Center, University of Missouri-Kansas City School of Medicine, Kansas City, USA

**Keywords:** seminoma, cancer associated retinopathy, sudden vision loss

## Abstract

Cancer-associated retinopathy (CAR) is a rare cause of vision loss that was first reported in 1976. It is reported that the retinopathy associated with cancer occurs due to antibodies against the tumor antigens that cross-react with retinal cell layers. We present the case of a young male who came to the emergency department with sudden onset of bilateral vision loss. He had a large-sized testicular seminoma with metastatic retroperitoneal lymphadenopathy. Several primary ophthalmological and systemic conditions were considered. He had multiple, positive anti-retinal antibodies. The cancer was felt to be the cause of the vision loss based on the clinical presentation and the presence of anti-retinal antibodies. He was treated with intravenous steroids, plasmapheresis, and curative chemotherapy, but there was no improvement in vision. Unfortunately, he died due to multiorgan failure. Our case is the second on seminoma-associated retinopathy in the literature.

## Introduction

Testicular cancer (TC) is the most frequent malignant neoplasm in young males aged 15 to 35 years [1]. Most cancers of the testis are of germ cell origin, which is divided based on the histological appearance into seminomas and non-seminomas. Testicular germ cell tumors usually present as a nodule or painless swelling of one testicle, which may be noted incidentally by the patient or the sexual partner. Metastatic disease is the first manifestation in around 10% of patients [2]. Vision loss in patients with TC is sporadic and is correlated with cancer-associated retinopathy (CAR). Barba-Navarrete et al. reported that around 47% of patients with TC had posterior segment alterations during an ophthalmological examination, out of whom only 4.7% had ocular symptoms [3]. We report the second case of vision loss from TC-associated retinopathy, with the first case being reported from Japan [4].

## Case presentation

A 35-year-old male with no medical history presented to our hospital with sudden onset bilateral vision loss that he noticed after waking up. He reported seeing wavy lines and abnormal colors two days before developing a complete loss of vision. He denied diplopia, redness, flashes, floaters, or difficulty with eye movement. He noted a two-year history of a mass in his scrotum associated with 20-pound unintentional weight loss and abdominal pain in the left lower quadrant for the previous three weeks. He denied seeking medical care previously for the scrotal mass due to a lack of insurance. He worked as a construction worker and denied drinking alcohol, using recreational drugs, or taking prescription medications. On the ophthalmological examination, there was no light perception, and pupils were dilated (9 mm) and sluggishly reactive to light. Sclera and conjunctiva were quiet, and intraocular pressure was 18 mm Hg bilaterally. The fundus examination showed flat optic disc with no edema, vitreous was clear, and macula was flat with no holes, tears, or blood bilaterally. The pelvic examination showed an enlarged right testis measuring around 10 cm in the largest dimension.

**Figure 1:**
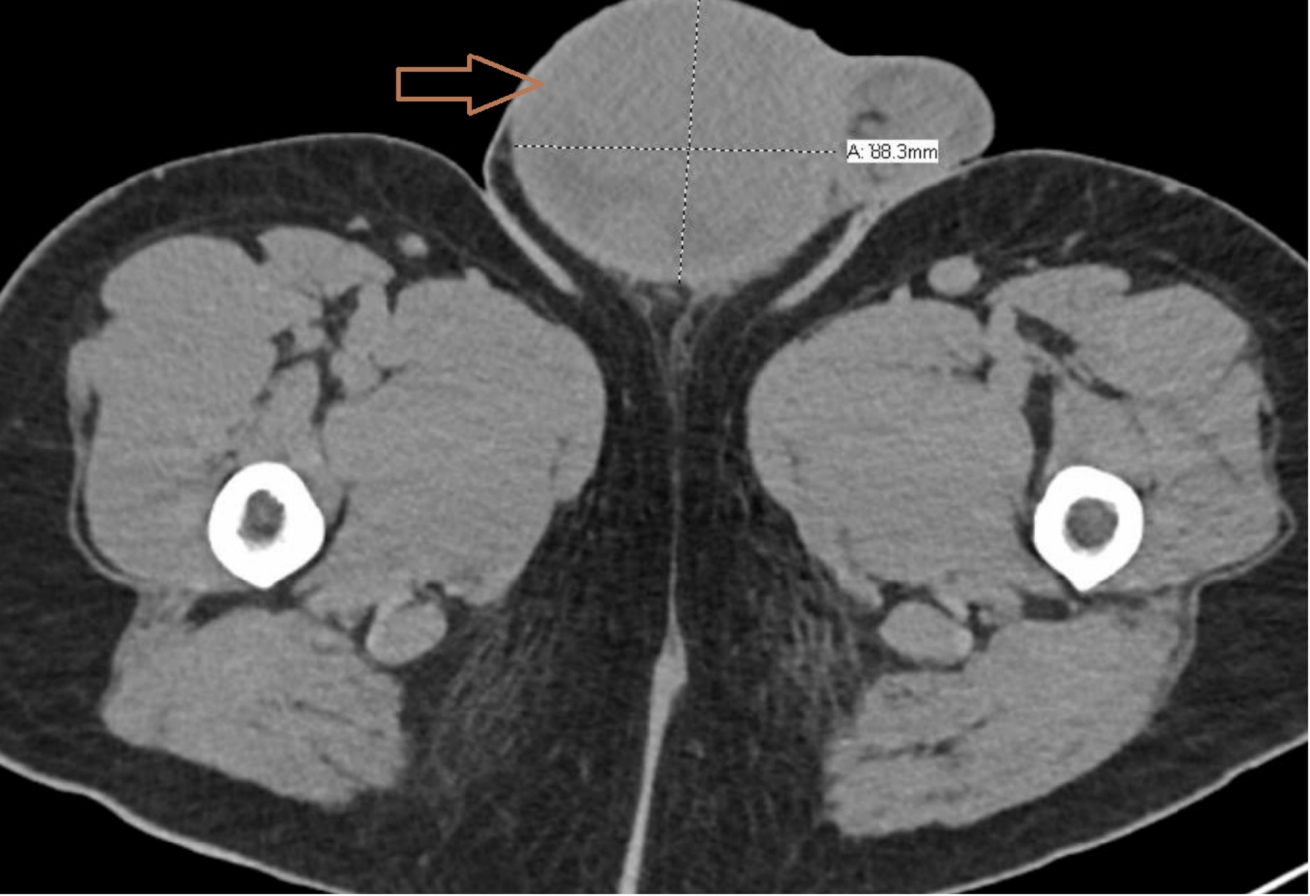
Right testicular solid mass of 8.1 cm x 8.1 cm size seen on CT scan (arrow) CT, computed tomography

**Figure 2:**
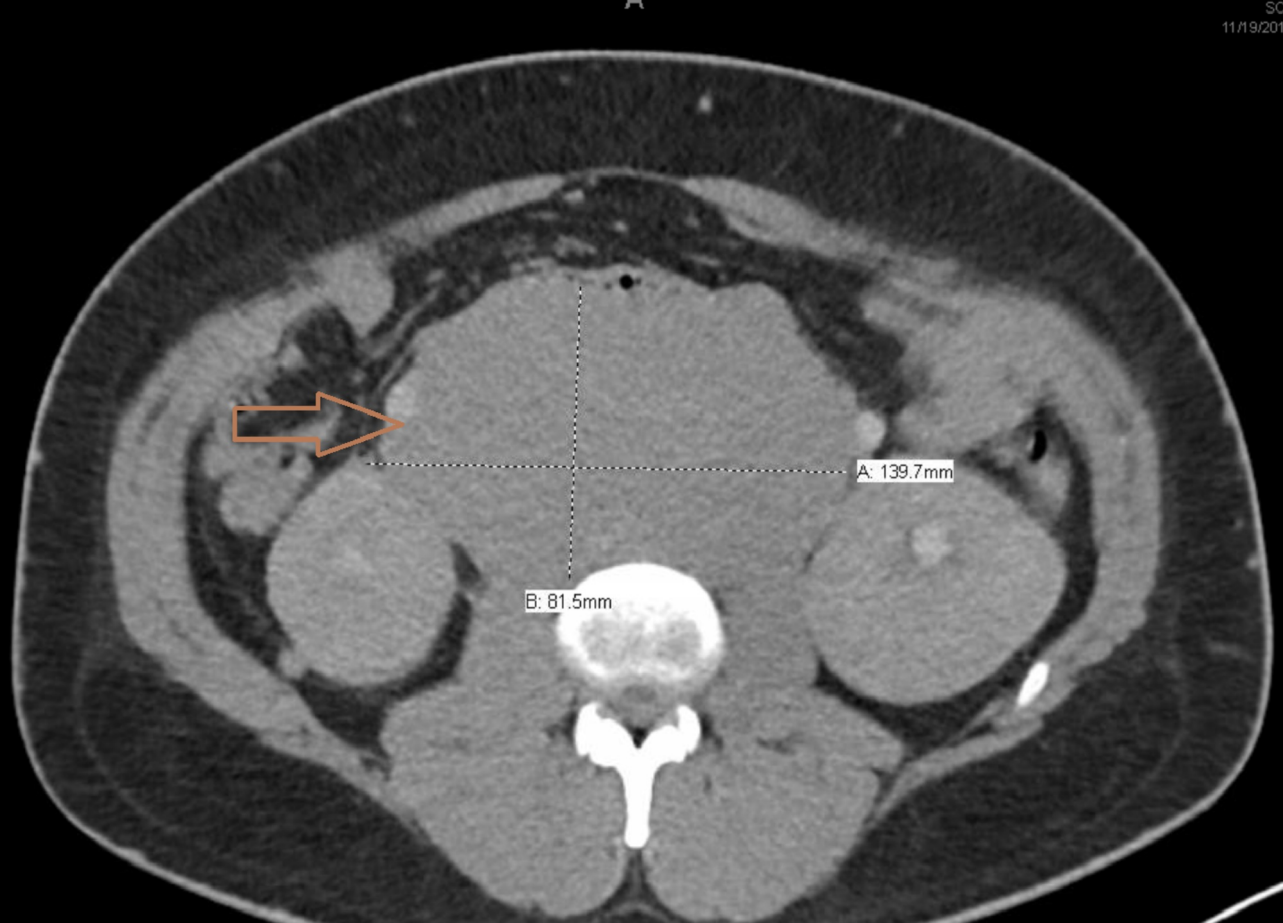
Large retroperitoneal lymphadenopathy measuring 18.1 cm x 14 cm x 8.2 cm in size (arrow)

Blood tests showed acute kidney injury with creatinine of 11 mg/dL (normal: 0.9 to 1.3 mg/dL) and metabolic acidosis with pH of 7.1 and serum bicarbonate of 14 mmol/L (normal: 22 to 32 mmol/L), but lactic acid was normal. The uric acid level was elevated at 11.2 mg/dL (normal: 4.8 to 8.7 mg/dL). Ethylene glycol and methanol levels were negative. Erythrocyte sedimentation rate (ESR) was high at 114 mm/hour (normal: 1 to 10 mm/hour). The anti-nuclear antibody (ANA) was positive in 1:640 titer with a positive anti-ribonucleoprotein antibody. Anti-neutrophil cytoplasmic antibody panel and serum protein electrophoresis were negative. Tumor markers revealed high lactate dehydrogenase (LDH) at 2,160 units/L (normal: 135 to 250 units/L). However, alpha-fetoprotein (AFP) and human chorionic gonadotrophin (HCG) levels were normal. Magnetic resonance imaging (MRI) of the brain and orbits were unremarkable. Computed tomography (CT) scan of the pelvis showed an 8.1 x 8.8 cm right testicular mass (Figure 1). CT scan of the abdomen showed an 18.1 x 14 x 8.2 cm mass in the retroperitoneum (Figure 2), which had calcification and internal hemorrhage. Mild bilateral hydronephrosis was present. Spinal fluid analysis, including bacterial cultures, viral polymerase chain reaction panel, and cytology, were negative. Core biopsy of the retroperitoneal mass showed tumor cells consistent with seminoma. We did not detect any immunophenotypic evidence of B- or T-cell lymphoma on flow cytometry.

Sudden loss of vision in a young man has several possible causes. In the emergency department, the patient was given fomepizole and high-dose intravenous (IV) methylprednisolone to treat potential methanol ingestion and ischemic arteritis, respectively. The ophthalmology team felt that retinal disease was the most likely cause of vision loss due to the normal slit-lamp eye examination (including fundal examination) and normal MRI of the brain and the orbits. They considered optical coherence tomography (OCT) but concluded that it would not change management given the overall clinical scenario. An electroretinogram (ERG) was not available at our facility. The rheumatology team opined that the patient was too young to develop temporal arteritis, as this is usually a disease of patients above 50 years of age. They also noted that the positive ANA test was possibly a red herring rather than a significant finding, as there was no response to steroids. We then considered various causes of retinal damage, including hereditary photoreceptor degeneration (e.g., cone dystrophy and retinitis pigmentosa), autoimmune/paraneoplastic retinopathy, toxic-nutritional (methanol, drugs) retinopathy, and infectious (e.g., acquired syphilis) etiologies. The time course of the patient's vision loss was suggestive of an acquired (weeks to months) rather than hereditary disease (months to years). The patient did not have a family history of vision loss and denied toxic medication use (drugs such as chloroquine/thioridazine). Infectious disease recommended spinal fluid analysis, which did not show any signs of infection, including the rapid plasma regain (RPR) test. After extensive workup, our top differential was CAR. The paraneoplastic anti-retinal antibody panel returned positive, and we found antibodies against retinal antigens: recoverin, carbonic anhydrase II, aldolase, alpha-enolase, glyceraldehyde 3-phosphate dehydrogenase, and ras-related protein-6. Immunohistochemistry of the human retina showed moderate staining of the photoreceptor cell, inner plexiform, and ganglion cell layers. Western blotting showed anti-retinal antibodies against 30 kDa, 35 kDa, 40 kDa, 46 kDa, and 58 kDa proteins. Although a positive anti-retinal antibody panel alone does not confirm the diagnosis of CAR, in the setting of a large metastatic seminoma and the absence of alternate etiologies, it was highly suggestive of the diagnosis of CAR.

High-dose steroids were continued for five days, and he received five plasmapheresis treatments. He underwent bilateral nephrostomy tube placement and temporary hemodialysis. Cancer staging showed stage 3 primary testicular seminoma. The oncology team noted that there was a high chance of cure (80% five-year survival rate) for advanced seminoma. One cycle of curative chemotherapy with bleomycin, etoposide, and cisplatin (BEP) was administered. Unfortunately, the patient's vision did not improve despite steroids, plasmapheresis, and chemotherapy. A week after cycle 1 of chemotherapy, he developed pancytopenia and septic shock from pyelonephritis. We held a family meeting to discuss goals of care, and the family decided to pursue comfort care. The patient passed away two days later.

## Discussion

**Figure 3:**
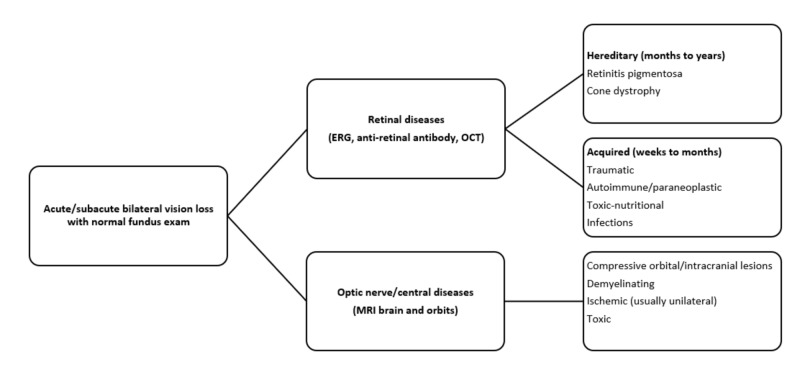
Evaluation of acute bilateral vision loss with normal fundus examination ERG, electroretinogram; OCT, optical coherence tomography; MRI, magnetic resonance imaging

Our case is the first report from the United States on seminoma-associated retinopathy. We highlight the stepwise approach to the evaluation of sudden vision loss with a normal fundus examination (Figure 3). CAR is the most common intraocular paraneoplastic syndrome. It was first reported in 1976 by Sawyer et al. [5]. Several cancers are known to cause this type of retinopathy, with small cell lung cancer and malignancies of the female reproductive organs and breast reported most commonly [6]. The underlying mechanism is molecular mimicry, which results in antibodies secreted against different cancer cells to target healthy retinal tissue [7]. The presence of anti-retinal antibodies is reported in around 65 % of patients with CAR [8]. Around 18 antigens are implicated, with the most common ones being recoverin (23 kDa) and alpha-enolase (46 kDa). Our patient tested positive for six out of the eight retinal antigens that were tested, including both recoverin and alpha-enolase.

The usual presentation of CAR is a painless subacute loss of vision that may predate the diagnosis of cancer or occur during or after treatment of the tumor. Symptoms are usually bilateral, as in our patient, but unilateral retinopathy is also reported [9]. Jacobson et al. proposed a clinical triad to diagnose CAR. It includes photosensitivity, ring scotomata, and attenuated arteriolar caliber [10]. However, our patient did not fulfill this triad due to complete vision loss. The fundus examination is variable, but it is sometimes unremarkable as in our patient. Findings, when present, include optic nerve pallor and attenuated retinal arterioles. Retinal pigment epithelium thinning and mottling are seen later in the disease course [11]. Ancillary tests for diagnosis include ERG and anti-retinal antibody panel. In our patient, CAR was thought to be the most likely diagnosis due to the clinical presentation, lack of another diagnosis, and the presence of anti-retinal antibodies.

Treatment of retinopathy due to cancer involves immunosuppression. Various therapies have been reported to be beneficial. These include IV and intraocular injections of steroids, IV immunoglobulin, and plasmapheresis. Some immunomodulatory agents such as azathioprine and mycophenolate are used as well. Reports exist of successful use of rituximab (anti CD 20 antibody) and alemtuzumab (anti CD 52 antibody) [12,13]. However, as in our patient, the prognosis for CAR is usually poor with progressive loss of visual acuity despite using immunosuppressive medications.

## Conclusions

Primary ophthalmological or systemic conditions can cause sudden onset vision loss. It is essential to pursue a thorough workup to rule out all possible etiologies. CAR is a challenging diagnosis that should always be considered in patients with underlying malignancies. A trial of empiric steroids should be given if the presentation is typical and infection has been ruled out. However, the prognosis for CAR is usually poor with progressive loss of vision.
